# Taurine Attenuates Oxidized Fish Oil-Induced Oxidative Stress and Lipid Metabolism Disorder in Mice

**DOI:** 10.3390/antiox11071391

**Published:** 2022-07-18

**Authors:** Qiuping Guo, Lingyu Zhang, Yunju Yin, Saiming Gong, Yuhuan Yang, Sisi Chen, Mengmeng Han, Yehui Duan

**Affiliations:** 1 Hunan Provincial Key Laboratory of Animal Nutritional Physiology and Metabolic Process, CAS Key Laboratory of Agro-Ecological Processes in Subtropical Region, National Engineering Laboratory for Pollution Control and Waste Utilization in Livestock and Poultry Production, Institute of Subtropical Agriculture, Chinese Academy of Sciences, Changsha 410125, China; guoqiuping@isa.ac.cn (Q.G.); zzzhanglingyu@163.com (L.Z.); yinyj1124@163.com (Y.Y.); 15197442484@163.com (S.G.); yangyu709842339@163.com (Y.Y.); 13574350026@163.com (S.C.); 15591808189@163.com (M.H.); 2 National Engineering Laboratory for Rice and By-Product Deep Processing, Central South University of Forestry and Technology, Changsha 410004, China; 3 College of Animal Science and Technology, Hunan Agricultural University, Changsha 410128, China; 4 College of Advanced Agricultural Science, University of Chinese Academy of Sciences, Beijing 100039, China

**Keywords:** oxidized fish oil, taurine, liver injury, oxidative stress, lipid metabolism

## Abstract

The objective of this study was to determine the effect of dietary taurine on lipid metabolism and liver injury in mice fed a diet high in oxidized fish oil. The ICR mice (six weeks old) were randomly assigned to six groups and fed different diets for 10 weeks: control (CON), normal plus 15% fresh fish oil diet (FFO), normal plus 15% oxidized fish oil diet (OFO), or OFO plus 0.6% (TAU1), 0.9% (TAU2) or 1.2% (TAU3) taurine. Compared to the CON group, OFO mice showed increased liver index, aspartate aminotransferase (AST) and malondialdehyde (MDA) levels in serum (*p <* 0.05). In addition, OFO mice had increased cholesterol (CHOL)/high-density lipoprotein cholesterol (HDL-C) and decreased HDL-C/low-density lipoprotein cholesterol (LDL-C) and n-6/n-3 polyunsaturated fatty acid (PUFA) ratio in serum (*p* < 0.05) compared with CON mice. Notably, dietary taurine ameliorated the liver index and AST and MDA levels in serum and liver in a more dose-dependent manner than OFO mice. In addition, compared to OFO mice, decreased levels of CHOL and ratio of CHOL/HDL-C and n-6 PUFA/n-3 PUFA in serum were found in TAU3-fed mice. Supplementation with TAU2 and TAU3 increased the relative mRNA expression levels of peroxisome proliferator-activated receptor α, adipose triglyceride lipase, lipoprotein lipase, hormone-sensitive lipase and carnitine palmitoyl transferase 1 in liver compared with the OFO group (*p* < 0.05). Moreover, impaired autophagy flux was detected in mice fed with the OFO diet, and this was prevented by taurine. These findings suggested that dietary taurine might provide a potential therapeutic choice against oxidative stress and lipid metabolism disorder.

## 1. Introduction

Currently, fish oil may have unlimited usage for its alleviation effects on obesity, nonalcoholic fatty liver disease and inflammation, which could reasonably be attributed to its rich content of n-3 polyunsaturated fatty acids (PUFAs), including docosahexaenoic acid (DHA) and eicosapentaenoic acid (EPA) [1,2]. However, fish oil is highly prone to lipid peroxidation under the promoting effect of air, light and heat because of a high double-bond content and unique position for double bonds within the fatty acid chain [3]. The peroxidation of PUFA is a complex process starting from lipid radical formation, which is particularly favorable because the lipid radical is resonance stabilized; lipid radicals can react with oxygen, and this reaction creates lipid peroxides, which in turn react with unoxidized PUFAs [3,4]. Thermal processing of PUFAs altered the fatty acid profile. In addition, lipid peroxides easily decompose to secondary products, including aldehydes such as malondialdehyde (MDA), 4-hydroxy-2(E)-nonenal, and 4-hydroxy-2(E)-hexenal. MDA is one of the most popular markers of lipid peroxidation. Previous studies demonstrated that many lipid peroxides and secondary oxidation products exceeded the recommended level in fish oil products on the market [5,6,7].

In the liver, fat accumulation and lipid peroxides are essential factors in producing liver injury [8]. Autophagy is a major intracellular recycling system that has shown positive effects in maintaining cell homeostasis. Previous studies have shown that autophagy regulates lipid metabolism [9] and oxidative stress [10]. Low levels of basal autophagy play a vital role in preventing the gradual accumulation of damaged proteins and organelles. A decline in autophagy flux caused a gradual decline in lysosomal clearance, which is relevant for accumulating reactive oxygen species under oxidative stress [11]. Impairment of autophagy flux has been observed in many chronic liver diseases, chronic viral hepatitis and nonalcoholic fatty liver disease [12,13].

Taurine (2-aminoethylsulfonic acid) is a type of β-amino acid and found in excitable tissues in approximately every animal tissue [14]. Numerous studies have investigated its physiological and pharmacological activity [14,15,16]. The anti-oxidative stress actions of taurine have been widely investigated [17,18]. It is reported that chronic treatment with taurine suppresses ROS accumulation in arsenic-treated myotubes [17]. In addition, taurine has been shown to inhibit lipid accumulation and improve lipid metabolism. Dietary taurine supplementation ameliorates high-fat diet-induced obesity as well as improving resting energy expenditure [19]. Furthermore, taurine has been shown to attenuate chronic inflammation in adipose tissue and retard insulin resistance [20]. Whereas the anti-oxidative and anti-obesity properties of taurine have been abundantly investigated, few studies have been devoted to investigating the protective effects of taurine on liver injury in mice subjected to oxidized fish oil as a model of oxidative stress and lipid metabolism disorder.

Hence, in this study, we sought to investigate the protective role of different dosages of taurine against oxidized fish oil-induced oxidative stress and lipid metabolism disorder in mice and to carry out some investigations on its potential mechanisms of action. The results of the present study might provide a nutritional strategy for preventing oxidative stress.

## 2. Materials and Methods

### 2.1. Animals and Diets

The animal handling protocol was approved by the Institute of Subtropical Agriculture Chinese Academy of Sciences Animal Care and Use Committee. A total of 60 male ICR mice (6 weeks) were purchased from SLAC Laboratory Animal Central (Changsha, China) and randomly divided into six groups. All animals had free access to food and drinking water and were housed in a temperature- and humidity-controlled room (temperature, 25 ± 2 °C; relative humidity, 55 ± 10%; lighting cycle, 12 h/d 07:00–19:00). All diets were provided by Huafukang Biological Technologies (Beijing, China). The supplemented fish oil was purchased from Rongcheng Haida Fish Meal Co., Ltd. (Shandong, China). Fresh fish oil was oxidized by heating at 80 °C with continuous bubbling air until the peroxide value (POV) changed from 3.7 to 250 meqO_2_/kg. The POV of oxidized oil was analyzed according to AOCS official method 965.33 (2007) [21]. Taurine was purchased from Zhangjiagang Specom Biochemical Co., Ltd. (Jiangsu, China). The mice were randomized into 6 groups for 10 weeks (*n* = 10/group): the normal control diet group (CON); the control diet plus 15% fresh fish oil group (FFO); the control diet plus 15% oxidized fish oil group (OFO, POV = 250 meqO_2_/kg); the OFO diet supplemented with 0.6% taurine (TAU1); the OFO diet supplemented with 0.9% taurine (TAU2); the OFO diet supplemented with 1.2% taurine (TAU3). All the diets were stored at −20 °C throughout this study until needed.

### 2.2. Serum Paraments

Serum aspartate transaminase (AST), alanine aminotransferase (ALT), triglycerides (TAG), cholesterol (CHOL), high-density lipoprotein cholesterol (HDL-C), and low-density lipoprotein cholesterol (LDL-C) were analyzed on the Roche Cobas (Roche Cobas 311, Roche Diagnostics, Basel, Switzerland). The malondialdehyde (MDA) kit, the superoxide dismutase (SOD) kit, and the glutathione peroxidase (GPX) kit were obtained from Nanjing Jiancheng Bioengineering Institute (Nanjing, China).

### 2.3. Fatty Acid Profile Analysis of Fish Oil and Serum

The total lipid of fresh fish oil and oxidized fish oil was extracted by the previous study [22] using petroleum ether–benzene (1:1, *v*/*v*). The upper phase was transferred and then transesterified with potassium hydroxide/methanol. For serum fatty acid profile analysis, the serum sample was mixed with acetyl chloride:methanol (5:100, *v*/*v*) and then subjected to methanolysis at 50 °C for 8 h. The fatty acid methyl esters were analyzed by gas chromatographer (Agilent 689N). The injector and detector port temperatures were set at 250 °C. The initial oven temperature was set at 45 °C for 1 min and then increased to 215 °C at 13 °C/min. Hydrogen was used as carrier gas. The fatty acid profile was quantified according to the peak area and expressed as a percentage based on total fatty acids. The fatty acid composition of fresh fish oil and oxidized fish oil was shown in Table 1.

### 2.4. Histological Analyses of the Liver

The parts of the liver were embedded in paraffin, and then tissue blocks were sliced in 5 μm thickness and stained with hematoxylin and eosin (H&E). Liver slides were observed under the microscope (Motic BA210; Motic Medical Diagnostic Systems, Co., Ltd., Xiamen, China).

### 2.5. Assay of Enzyme Activity in Liver

Frozen liver tissues were homogenized in ice-cold PBS buffer and centrifuged at 3000 rpm for 10 min at 4 °C. The concentrations of MDA, AST, ALT and TAG in liver were measured according to the manufacturers (Nanjing Jiancheng Bioengineering Institute, Nanjing, China).

### 2.6. Relative mRNA Expression in Liver

Total RNA was extracted from the frozen liver tissue using the Trizol reagent (Invitrogen, Carlsbad, CA, USA), and reverse transcription was performed according to the manufacturer’s instructions of PrimeScript RT reagent Kit (Takara, Dalian, China). The mRNA levels were quantified using a real-time PCR system (Roche Diagnostics, Indianapolis, IN, USA) with SYBR Premix EX TaqⅡ (Takara, Dalian, China). PCR conditions were as follows: 95 °C for 10 min for initiation, followed by 40 cycles of 5 s at 95 °C, 30 s at 60 °C. Primers for the target genes were characterized in Table 2. Relative expression of the target gene was normalized to the expression of β-actin by the 2^−ΔΔCt^ method.

### 2.7. Western Blotting

Frozen samples of liver were crushed into powder in liquid nitrogen and homogenized. Protein concentration was determined by using the BCA protein assay kit (P0010-1, Beyotime, China). Lysates taken from each sample were separated by 12.5% SDS-PAGE. They were immunoblotted with primary antibodies against LC3, QSTM1/p62, and tubulin (2775 s, 23214 s and 2148 s, Cell Signaling Technology, Boston, MA, USA) overnight at 4 °C, and then incubated with horseradish peroxidase (HRP)-conjugated secondary antibodies (Protein Tech Group Inc., Chicago, IL, USA). The expected protein bands were visualized using the Quantity One software (Bio-Rad, Hercules, CA, USA).

### 2.8. Statistical Analyses

Data are expressed as the means ± SEM. The data were analyzed using one-way ANOVA, SAS 8.2 (SAS Institute, Inc., Cary, NC, USA), and graphs were generated using GraphPad Prism 8.2.1(GraphPad Software Inc., San Diego, CA, USA). Differences between significant mean values were compared using Duncan’s multiple range test. In the case of a *p* value < 0.05, differences were statistically significant.

## 3. Results

### 3.1. Taurine Decreased the Liver Index in OFO-Treated Mice

**Figure 1:**
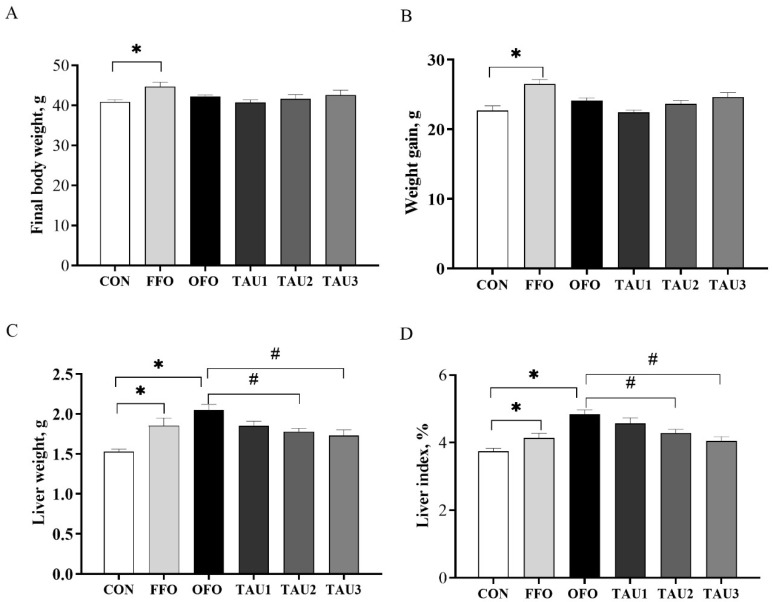
The final body weight (**A**), weight gain (**B**), liver weight (**C**) and liver index (**D**) of all groups of mice. ICR mice were fed a normal (CON), normal plus fish oil diet (FFO), normal plus oxidized fish oil diet (OFO), or OFO plus 0.6% (TAU1), 0.9% (TAU2) or 1.2% (TAU3) taurine diet for 10 weeks. Each value represents the mean ± SEM (*n* = 10). * *p* < 0.05 vs. the CON group and # *p* < 0.05 vs. the OFO group.

At the end of the experimental period, FFO-fed mice showed a higher body weight (Figure 1A; Figure S1, Supplementary Materials). Compared with mice in the CON group, OFO-fed mice showed a higher liver index (*p* < 0.05; Figure 1D), but showed no significant difference in final body weight, weight gain and liver weight (*p* > 0.05; Figure 1B–D). Taurine supplementation did not alter final body weight and weight gain but decreased liver weight and the liver index in a dose-dependent manner compared with the OFO group (Figure 1C,D).

### 3.2. Taurine Decreased Liver Injury in OFO-Treated Mice

**Figure 2:**
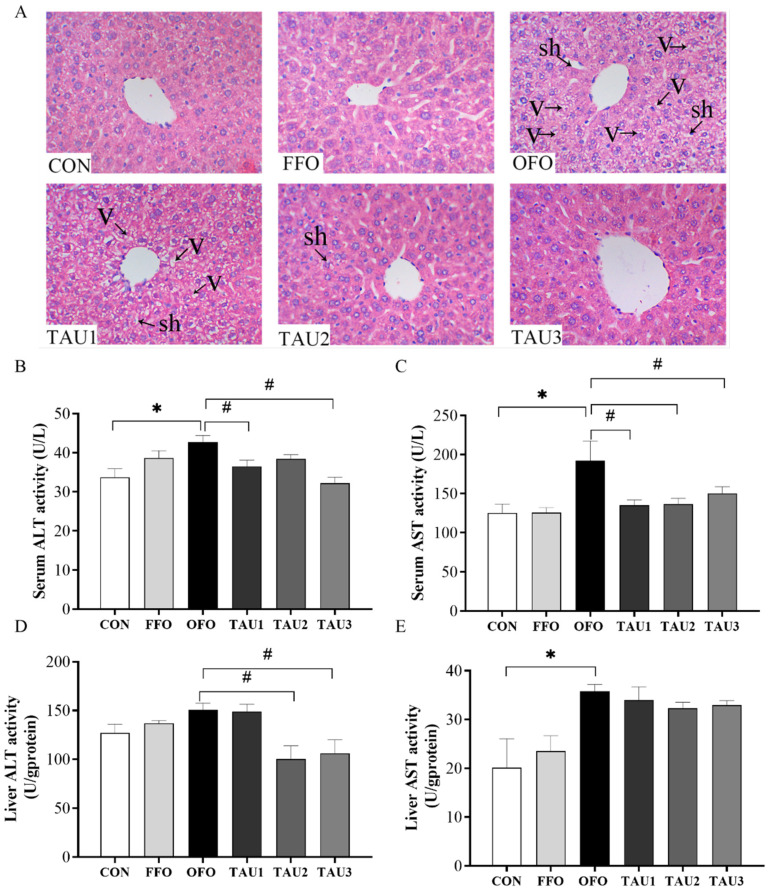
The liver histological findings ((**A**), hematoxylin and eosin staining, Vacuolization (v), swelling of hepatocytes (sh), 400×), serum ALT (**B**), serum AST (**C**), liver ALT (**D**) and liver AST (**E**) of all groups of mice. ICR mice were fed a normal (CON), normal plus fish oil diet (FFO), normal plus oxidized fish oil diet (OFO), or OFO plus 0.6% (TAU1), 0.9% (TAU2) or 1.2% (TAU3) taurine diet for 10 weeks. Each value represents the mean ± SEM (*n* = 10). * *p* < 0.05 vs. the CON group and # *p* < 0.05 vs. the OFO group. ALT, alanine aminotransferase; AST, aspartate transaminase.

As shown in Figure 2A, OFO supplementation led to damaged spatial structure, cellular vacuolization, and swelling of hepatocytes. Taurine supplementation suppressed the alteration of the liver structure, which showed a dose-dependent effect. To further examine the alleviative effects of taurine on liver injury, we tested AST and ALT activities in serum and liver. A diet with OFO increased the AST activity compared with the CON group (*p* < 0.05; Figure 2C,E). The enzyme activity of serum AST was significantly decreased with taurine supplementation compared with the OFO group (*p* < 0.05; Figure 2C). A 1.2% taurine supplementation reduced the elevation of ALT activities both in serum and liver (*p* < 0.05; Figure 2B,D).

### 3.3. Taurine Decreased Oxidative Stress and Liver Injury in OFO-Treated Mice

**Figure 3:**
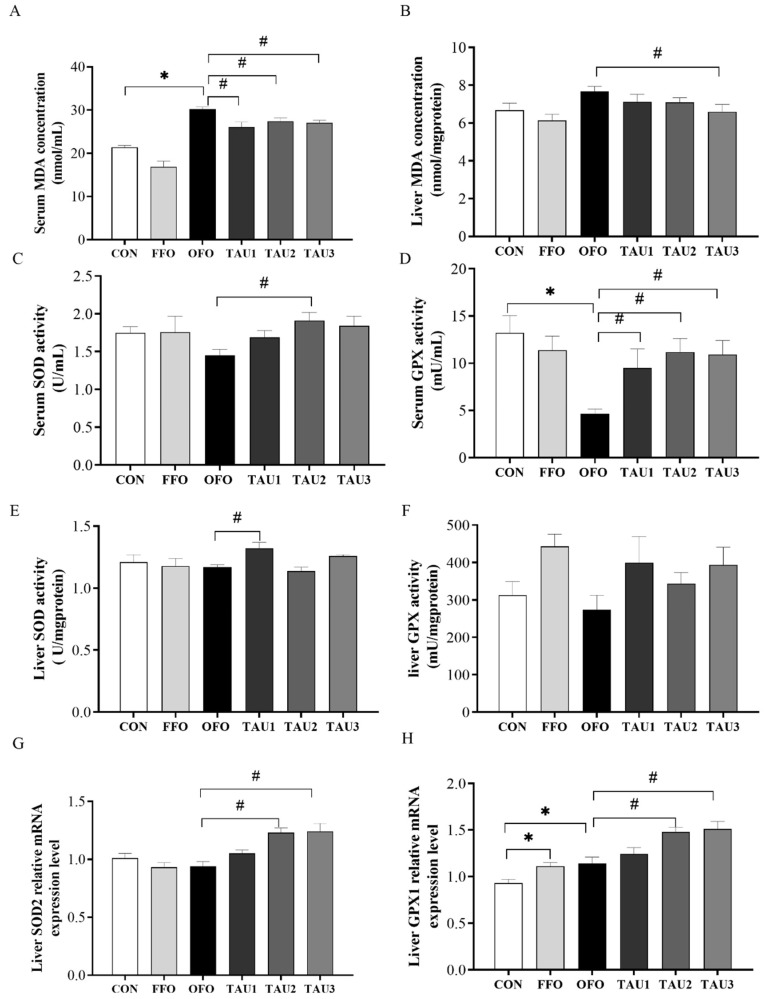
The serum MDA (**A**), liver MDA (**B**), serum SOD (**C**), serum GPX (**D**), liver SOD (**E**), liver GPX (**F**), relative mRNA expression levels of SOD2 (**G**) and GPX1 (**H**) in liver of all groups of mice. ICR mice were fed a normal (CON), normal plus fish oil diet (FFO), normal plus oxidized fish oil diet (OFO), or OFO plus 0.6% (TAU1), 0.9% (TAU2) or 1.2% (TAU3) taurine diet for 10 weeks. Each value represents the mean ± SEM (*n* = 10). * *p* < 0.05 vs. the CON group and # *p* < 0.05 vs. the OFO group. MDA, malondialdehyde; SOD, superoxide dismutase; GPX, glutathione peroxidase.

To confirm oxidative stress in mice following OFO intake, we measured the concentration of MDA in serum and liver. Mice fed the OFO diet showed significantly increased MDA concentrations in serum compared with the CON group (*p* < 0.05; Figure 3A). Taurine supplementation decreased MDA concentration in serum (*p* < 0.05; Figure 3A); 1.2% taurine treatment decreased MDA concentration in liver (*p* < 0.05; Figure 3B). Antioxidant enzymes, including SOD and GPX, play a vital role in defending oxidative status in animals. In this study, we measured the SOD and GPX activities in serum and liver and relative mRNA expression levels in the liver. Supplementation with taurine increased serum GPX activity compared with the OFO group (*p* < 0.05; Figure 3D). Supplementation with 0.9% or 1.2% increased SOD2 and GPX1 relative mRNA expression levels in liver (*p* < 0.05; Figure 3G,H).

### 3.4. Taurine Altered Serum Lipid Parameters in OFO-Treated Mice

As shown in Table 3, compared with the FFO groups, 10 weeks of OFO exposure significantly increased the level of CHOL, LDL-C, the ratio of CHOL/HDL-C, and decreased the ratio of HDL-C/LDL-C in serum of mice (*p* < 0.05). Meanwhile, the level of TAG in liver was significantly higher in the OFO group than in the CON group (*p* < 0.05). A 1.2% taurine supplementation suppressed the elevation of TAG and CHOL concentration in serum and increased TAG levels in the liver compared with the OFO group (*p* < 0.05). Taurine supplementation increased the HDL-C/LDL-C ratio and suppressed the elevation of serum LDL-C levels in a dose-depend manner (*p* < 0.05); the serum HDL-C levels did not differ among the three taurine supplementation groups.

### 3.5. Taurine Changed Serum Fatty Acid Profile in OFO-Treated Mice

As shown in Table 4, serum fatty acid profile was significantly altered by dietary fresh fish oil or oxidized fish oil. The long-chain PUFA proportions of C18:3n3 and C22:6n3 were significantly increased while C18:2n6c and C20:4n6 significantly decreased in the serum of FFO compared to the CON group (*p* < 0.05). Elevated proportions of the most predominate SFA (C16:0) were discovered in mice fed the OFO diet. The proportions of C20:3n6 and C22:6n3 in 1.2%taurine supplemented groups were higher than in the OFO group, the proportions of C20:4n6 and C24:0 in taurine supplemented groups were lower than in the OFO group. Moreover, dietary taurine supplementation reduced the ratio of n-6/n-3 compared to the OFO treatment (*p* < 0.05).

### 3.6. Taurine Improved Lipid Metabolism in Liver in OFO-Treated Mice

**Figure 4:**
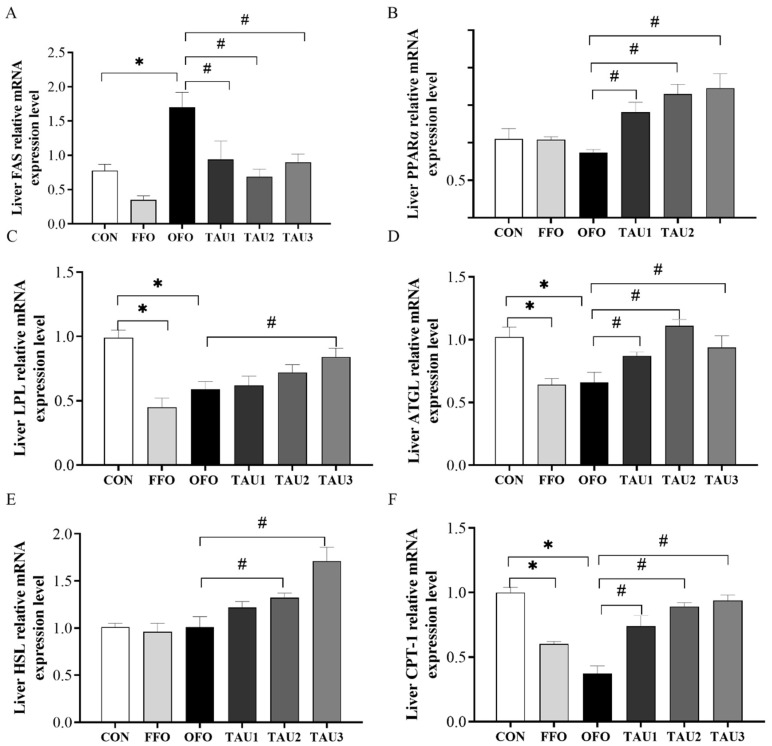
The relative mRNA expression levels of FAS (**A**), PPARα (**B**), LPL (**C**), ATGL (**D**), HSL (**E**), CPT-1 (**F**) in liver of all groups of mice. ICR mice were fed a normal (CON), normal plus fish oil diet (FFO), normal plus oxidized fish oil diet (OFO), or OFO plus 0.6% (TAU1), 0.9% (TAU2) or 1.2% (TAU3) taurine diet for 10 weeks. Each value represents the mean ± SEM (*n* = 10). * *p* < 0.05 vs. the CON group and # *p* < 0.05 vs. the OFO group. FAS: fatty acid synthase; PPARα: peroxisome proliferator-activated receptor α; LPL: lipoprotein lipase; ATGL: adipose triglyceride lipase; HSL hormone-sensitive lipase; CPT-1, carnitine palmitoyl transferase 1.

To gain further insight into the alleviative effect of taurine on lipid metabolism in OFO-treated mice, we evaluated the expressions of lipometabolic genes. OFO elevated gene expression of hormone-sensitive lipase (FAS) (*p* < 0.05; Figure 4A). Mice fed with taurine significantly increased the relative mRNA expression levels of peroxisome proliferator-activated receptor α (PPARα), adipose triglyceride lipase (ATGL) and carnitine palmitoyltransferase 1 (CPT-1) in liver compared with the OFO group (*p* < 0.05; Figure 4B,D,F). Supplementation with 0.9% and 1.2% taurine increased the relative mRNA expression levels of lipoprotein lipase (LPL) and hormone-sensitive lipase (HSL) in liver compared with the OFO group (*p* < 0.05; Figure 4C,E). However, the relative mRNA expression level of FAS was significantly decreased in mice with taurine intake (*p* < 0.05; Figure 1A).

### 3.7. Taurine Regulated Autophagy Flux in OFO-Treated Mice

**Figure 5:**
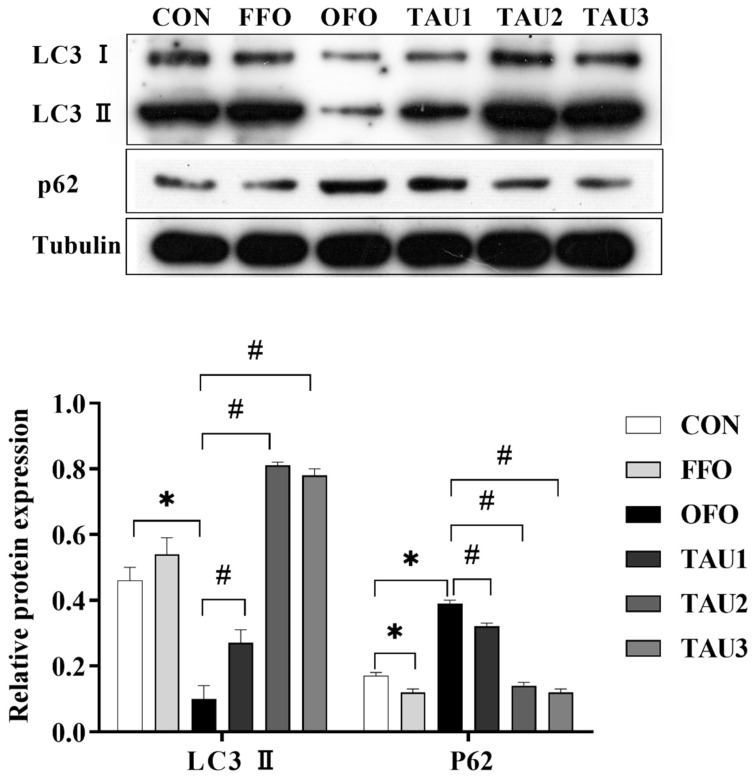
The relative expression level of autophagic-associated proteins in liver of mice of all groups. ICR mice were fed a normal (CON), normal plus fish oil diet (FFO), normal plus oxidized fish oil diet (OFO), or OFO plus 0.6% (TAU1), 0.9% (TAU2) or 1.2% (TAU3) taurine diet for 10 weeks. Each value represents the mean ± SEM (*n* = 10). * *p* < 0.05 vs. the CON group and # *p* < 0.05 vs. the OFO group.

To investigate whether oxidative stress alters autophagic flux in liver, Western blot analysis was used to detect the protein expression of LC3Ⅱ and p62. The results showed that the downregulation of LC3Ⅱ and upregulation of p62 relative expression caused by OFO treatment was relieved in mice fed a taurine diet (*p* < 0.05; Figure 5).

## 4. Discussion

Fish oil has long been recommended as a source of n-3 PUFA for its high content of eicosapentaenoic acid (EPA) and docosahexaenoic acid (DHA). Epidemiological investigations show that fish oil intake has an alleviative effect on metabolic syndrome [23,24]. However, fish oil is vulnerable to oxidative stress because of its high degree of unsaturation. Long-time intake of oxidized oil is associated with oxidative stress, which is now recognized as a major factor in the pathogenesis of liver injury and lipid metabolism disorder [25,26]. Some studies indicate that taurine has a significant anti-oxidation activity [14,27]. Therefore, we focused on the taurine alleviative effects against oxidative stress and lipid metabolism disorder induced by oxidized fish oil intake in the liver.

It has been well reported that oxidized oil decreases final body weight because oxidized oil products could be absorbed in the intestine and reduce nutrient absorption [28,29]. However, in this study, after the ten-week ad libitum feeding with OFO, the final body weight of mice did not alter compared with the fresh fish oil group. Our result corresponds with Lin and colleagues, who reported that dietary body weight did not differ with dietary oxidized oil intake [25].

Assessment of liver injury is based on the liver index, pathological changes in liver and liver homogenate contents, wherein the liver index is used as an indicator of susceptibility. That is because the liver is related closely with nutrient metabolism and subjected to oxidative stress [30]. In our study, feeding diets supplemented with oxidized fish oil increased the liver index compared with fresh fish oil intake, which is consistent with those reported in other studies [28,31]. The enlarged liver in pigs subjected to dietary oxidative stress was likely due to the cytotoxic effects of lipid oxidation products [30]. In the previous finding, 5% (*w/v*) taurine supplementation has been found to have anti-oxidation effects and suppress the elevation of liver weight [27]. In our study, supplementation with 0.9% or 1.2% taurine to mice decreased liver weight and the liver index, which means that taurine intake might be an effective therapy for inhibiting oxidative stress. AST and ALT are two main aminotransferases synthesized inside liver cells and are released to blood when the liver undergoes injury [32]. Chronic consumption of oxidized oil or high-fat diet-induced significant elevation of AST and ALT in pigs and mice [30,33]. In the current study, the liver injury-sensitive biomarker AST level was significantly increased in mice fed an OFO diet. Moreover, taurine supplementation significantly decreases the serum AST concentration in a non-dose-dependent manner.

The concentration of MDA provided further confirmation that oxidative stress occurred with OFO treatment. MDA, generated only through the peroxidation of PUFAs, has longer half-livers than ROS and diffusion characteristics, thereby expanding the influence of oxidative stress [34]. An elevated serum MDA concentration indicated lipid peroxidation significantly increased with fried oil intake in mice [35]. In our study, the concentration of MDA in serum and liver tissue was elevated in mice fed an OFO diet. The increase in MDA level in mice provided oxidized fish oil agrees with the liver injury indicator enzyme AST change. These facts, taken together, clearly indicated that the occurrence of liver injury with oxidized oil treatment and taurine treatment alleviated the oxidative stress in mice fed with oxidized oil. Chronic exposure to oxidative stress also alters the antioxidant defense system in liver tissue. SOD and GPX represent essential components of the antioxidant defense system involved in ROS-scavenging processes. Research examining the role of oxidized oil on the antioxidant system has produced mixed results. Several documents have indicated that the activities of antioxidant enzymes were stimulated by the oxidized oil feeding [36,37]. However, the GPX activities in serum and liver of mice fed the oxidized oil were significantly reduced in this study. Similar results have indicated that oxidized oil consumption could decrease the activity of enzymic antioxidants SOD, GPX and catalase in the liver of oxidized oil-fed individuals [25,38]. Taurine has been found to increase the antioxidant enzyme activities in hepatic [39,40]. The strong potential as an antioxidant for taurine was based on its ability not only to scavenge free radicals directly but also to prevent changes in membrane permeability induced by oxidant injury [41,42,43]. In our study, after taurine treatment, the activities of GPX increased in serum, an effect correlated with the increase in the expression of the SOD and GPX in liver. In the study, taurine might prevent GPX consumption through scavenging free radicals in mice fed with oxidized oil. Overall, the liver function indicators AST and ALT, oxidative stress markers MDA, SOD and GPX confirmed that feeding oxidized fish oil induces liver injury and taurine alleviates the injury in mice.

The previous study demonstrated that oxidative stress was positively correlated with lipid accumulation [44]. Hence, we observed serum lipid paraments, liver TAG concentration and related gene expression in this study. The effect of feeding diets with oxidized oil was well reported in the serum and liver of rodents [35,45,46]. However, some rather conflicting data exist about CHOL level in serum of oxidized oil-fed animals [35,46,47,48,49,50]. In animals fed oxidized fat, CHOL concentrations were decreased [47], not alternated [35,48], or even increased compared with controls fed fresh oil [46,49,50]. Our study shows increased levels of CHOL in the serum in mice fed with oxidized oil compared with the control group and fresh oil intake group. It has been indicated that HDL mediates the transport of excess CHOL from peripheral tissues to the liver, while LDL transports CHOL from the liver to peripheral tissues [51]. Therefore, CHOL/HDL-C ratio and HDL-C/LDL-C ratio could be regarded as markers that determine CHOL transport. In this study, mice fed the oxidized oil diets showed increased serum CHOL/HDL-C ratio and decreased HDL-C/LDL-C ratio, which suggested decreased carry CHOL ability from peripheral tissue to the liver. The results correspond with Murakami and colleagues, who reported that taurine decreased the ratio of CHOL/HDL-C in the serum of mice [27]. Decreased HDL-C/LDL-C ratio is associated with the risk of developing atherosclerosis. Taurine decreased CHOL/HDL-C ratio and increased the HDL/LDL-C ratio in a dose-depend manner that may hasten the removal of cholesterol from peripheral tissues to the liver for catabolism and excretion.

PUFAs are labile compounds that undergo peroxidative damage under the promoting effect of light, oxygen, or high temperature. Especially, n-3 PUFAs are more susceptible to lipid oxidation due to double bonds and their position within the fatty acid chain. In the study, the fatty acid profile of the oxidized fish oil was significantly changed compared with fresh fish oil. In general, thermal processing the fish oil decreased oil quality, including an increased ratio of SFA/UFA and n-6/n-3, and reduced percentage of C22:6n3 (DHA). The serum fatty acid profile is closely related to the dietary fatty acid profile. Previous study indicated that a diet with DHA and EPA significantly altered the fatty acid profile of the brain and blood in mice [52]. In the study, we found that OFO diet increased the ratio SFA/UFA and n-6/n-3 compared with the FFO group. The finding is in line with our previous study’s results, which showed that supplementation with oxidized fish oil decreased the ratio of n-6/n-3 in longissimus dorsi muscle compared with fresh fish oil [53]. Taurine has been investigated for its effects on lipid metabolism. The taurine intake improved the contention of C22:6n3 and reduced the ratio of n-6/n-3 in serum compared with OFO pigs in the study. We hypothesize that the antioxidant effects might be one reason for the higher proportion of C20:4n6 and C22:6n3 observed in the serum of TAU compared to OFO pigs. The results were supported by Lu who reported that the proportion of long chain unsaturated fatty acids, of backfat was significantly increased by the dietary vitamin E [54]. Furthermore, taurine, combined with EPA and DHA-rich fish oil, exhibits preventive effects on white adipose tissue weight gain and hyperglycemia in mice [55]. In addition, it was observed positive effects of taurine in combination with n-3 (EPA + DHA) in the blood of healthy humans [56]. Thus, improved serum lipid metabolism in TAU groups in the study could be partially attributed the antioxidant effects and additive effect of n-3 fatty acids.

To further investigate the beneficial role of taurine in liver lipid metabolism, we detected the gene expression related to lipid metabolism. FAS is an essential rate-controlling enzyme involved in fatty acid synthesis [57]. Overexpression of FAS may induce an increase in the development of fatty liver and PPAR-α agonism could reduce the elevation of FAS mRNA expression [58]. In our study, after 10 weeks of oxidized oil exposure, the gene expression level of FAS was significantly higher than that in the CON and FFO groups. Dietary taurine supplementation suppressed the elevation of FAS gene expression in the oxidized oil-stressed mice. Downregulation of FAS expression by taurine feeding could decrease TAG concentration and ectopic fat deposition in liver [15,59]. PPARα is a ligand-activated nuclear receptor and is highly expression in liver where it regulates genes involved in hepatic lipid metabolism, including lipolysis, lipogenesis and fatty acid catabolism [60]. The activation of PPAR-α ameliorated hepatic steatosis and insulin resistance; in contrast, disruption of PPAR-α signal in mice fails to meet energy demands and induces fatty liver [58]. This study observed a significant increase in PPARα expression level in the liver after being supplemented with taurine. Similar to our findings, drinking a taurine solution could upregulate PPARα and shows preventive effects on the development of hepatic steatosis in high-fat/CHOL -fed hamsters [61]. LPL is the key enzyme that catalyzes the hydrolysis of lipoprotein TAG into free fatty acid. It is thought that direct binding to a PPAR response element, or by decreasing expression and secretion of LPL inhibitor, PPARα controlled LPL levels [60]. It was observed that decreased LPL expression seems to induce dyslipidemia as ApoE−/− mice had reduced levels of HDL-C and elevated levels of TAG as well as CHOL [62]. Taurine was found to increase LPL expression in rats [63] efficiently. Coincidently, our data showed that taurine increased LPL gene expression in a dose-dependent manner in OFO-fed mice. Furthermore, HSL and ATGL are key rate-limiting hydrolases and play a vital role in liver lipolysis [64]. A study conducted in ATGL−/− mice indicated that ATGL regulates TAG turnover and the expression of PPARα-dependent genes [65]. Overexpression of hepatic HSL might benefit liver function as the animals coupled with reduced AST and ALT activity in plasma [66]. Moreover, overexpression of hepatic HSL and ATGL activates PPARα-targeted fatty acid oxidation gene expression and improves hepatic steatosis [60]. Taurine supplementation enhanced the expression of HSL and ATGL in this study, which indicated that taurine could reduce fat deposition in the liver. The CPT-1 enzyme is localized in the mitochondria membrane and involved with fatty acid transport to mitochondria for β-oxidation. CPT-1 is upregulated in the liver by PPARα activation [60,67]. Correspondingly, dietary OFO supplementation reduced the relative expression of CPT-1, while taurine suppressed the reduction dose dependently. Overall, the transcription study of genes involved in lipid metabolism in liver, including PPARα, FAS, LPL, ATGL, HSL and CPT-1, indicated that taurine could ameliorate lipid metabolic disorders by inhibiting fat synthesis, facilitating fat hydrolysis and fatty oxidation via regulated PPARα action in oxidized oil-fed mice.

Mounting evidence suggests a fundamental role for autophagy, the primary regulator of both the innate and adaptive intracellular recycling system that maintains cellular homeostasis under basal conditions and plays a vital role in protection against oxidative stress [10]. The LC3 protein is associated with autophagosome membranes, which could be cleaved explicitly at the C terminus by Atg4 to become LC3-I and then conjugates to phosphatidylethanolamine to form LC3-II. Since LC3-II could be degraded in autolysosomes, the levels of LC3-II are used as a marker for monitoring the autophagic process. The p62 protein is a link between LC3 and is degraded explicitly in autophagy [13,68]. Thus, the upregulated levels of LC3-II are associated with increased autophagosome formation, and the decreased p62 protein levels are associated with positive autophagy flux. Previous studies have found that taurine inhibited ROS generation and alleviated As_2_O_3_-induced autophagy [17,69]. OFO treatment resulted in increased LC3Ⅱ expression and autophagy adaptor p62 accumulation, indicating impaired autophagic flux in the liver, which was reversed by taurine treatment.

In conclusion, with the development of living levels and lifestyle alteration, fish oil and other nuts oil are extensively recommended as the source of n-3 PUFAs [1,70]. However, some studies indicated that a part of fish oil in the market exceeded the limit for primary oxidation [5,6,7]. Thus, combining powerful antioxidants and n-3 PUFAs might have significant beneficial effects. Our study provides evidence that taurine supplementation exerts antioxidants and attenuated lipid metabolic disorders effect on oxidized fish oil-fed mice. These findings led us to hypothesize that taurine could serve as a healthy additive in fish oil dietary supplements. Moreover, some polyphenols exhibit potent antioxidant and anti-inflammatory effects, allowing them to show synergistic, additive, or complementary effects with fish oil [71,72,73]. Further work is needed to determine how these nutraceuticals interact with each other in vivo and their primary mechanisms of action.

## 5. Conclusions

The above finding leads us to infer that oxidized fish oil induces oxidative stress, lipid metabolism disorder and blocked autophagy flow, which could be alleviated by taurine treatment. Taurine could be a potential natural ingredient to reduce the risk of liver injury and lipid metabolism disorder in animal and human nutrition.

## Figures and Tables

**Table 1:** Fatty acid composition of oxidized fish oil and fresh fish oil (% of total).

Items	Fresh Fish Oil	Oxidized Fish Oil
C14:0	9.46	10.24
C16:0	24.71	28.77
C16:1	10.88	9.40
C17:0	1.38	1.90
C18:0	4.91	5.58
C18:1n9t	0.19	0.21
C18:1n9c	16.90	15.34
C18:2n6c	3.23	2.86
C20:0	0.95	1.02
C18:3n6	0.29	0.21
C20:1	3.10	4.63
C18:3n3	1.97	2.23
C20:3n6	0.20	0.14
C20:4n6	1.44	1.19
C22:6n3	20.39	16.28

**Table 2:** Primers used for real-time PCR.

Gene	Sequence	Size (bp)	GeneBank No.
SOD2	TTCTGGACAAACCTGAGCCCTAA GAACCTTGGACTCCCACAGACAC	134	NM_013671.3
GPX1	AGGAGAATGGCAAGAATGAAGAGA GGAAGGTAAAGAGCGGGTGAG	135	NM_001329528.1
FAS	TGGTGAATTGTCTCCGAAAAGA CACGTTCATCACGAGGTCATG	149	AF127033
PPARα	ATCCCATCACTCTCTCTGTG AACTACCTGCTCAGGACTCA	161	NM_011144.6
LPL	CTGCTGGCGTAGCAGGAAGT GCTGGAAAGTGCCTCCATTG	231	NM_008509.2
ATGL	ATTTATCCCGGTGTACTGTG GGGACACTGTGATGGTATTC	119	XM_021167897.2
HSL	GTGAATGAGATGGCGAGGGT GTGCCCTCACAGCAGGAATA	101	NM_010719.5
CPT-1	AGCACACCAGGCAGTAGCTT AGGATGCCATTCTTGATTCG	144	NM_009948
β-actin	TCTTTTCCAGCCTTCCTTCTTG GAGGTCTTTACGGATGTCAACG	100	NM_007393

SOD2: superoxide dismutase; GPX1: glutathione peroxidase 1; FAS: fatty acid synthase; PPARα: peroxisome proliferator-activated receptor α; LPL: lipoprotein lipase; ATGL: adipose triglyceride lipase; HSL hormone-sensitive lipase; CPT-1, carnitine palmitoyl transferase 1.

**Table 3:** Lipid parameters in all experimental groups of mice ^1^.

Items	CON	FFO	OFO	TAU1	TAU2	TAU3
**Serum**						
TAG, mmol/L	1.49 ± 0.07	1.05 ± 0.08 *	1.46 ± 0.10	1.23 ± 0.10	1.25 ± 0.08	1.15 ± 0.07 ^#^
CHOL, mmol/L	3.24 ± 0.10	2.76 ± 0.11 *	4.03 ± 0.26 *	3.55 ± 0.11 ^#^	3.75 ± 0.14	3.25 ± 0.12 ^#^
LDL-C, mmol/L	0.33 ± 0.01	0.23 ± 0.01 *	0.41 ± 0.02 *	0.38 ± 0.03	0.35 ± 0.03	0.27 ± 0.02 ^#^
HDL-C, mmol/L	2.77 ± 0.08	2.68 ± 0.15	3.00 ± 0.13	3.16 ± 0.10	3.18 ± 0.18	3.16 ± 0.15
CHOL/HDL-C	1.17 ± 0.01	1.03 ± 0.03	1.34 ± 0.10 *	1.12 ± 0.01 ^#^	1.18 ± 0.02 ^#^	1.03 ± 0.04 ^#^
HDL-C/LDL-C	8.29 ± 0.43	11.70 ± 0.56 *	7.28 ± 0.35 *	8.38 ± 0.43	9.16 ± 0.46 ^#^	11.72 ± 0.44 ^#^
**Liver**						
TAG, mmol/g protein	37.73 ± 1.99	38.47 ± 1.87	48.65 ± 1.80 *	41.57 ± 3.63	41.70 ± 2.80	34.95 ± 2.47

* *p* < 0.05 vs. the CON group and # *p* < 0.05 vs. the OFO group. ^1^ ICR mice were fed a normal (CON), normal plus fish oil diet (FFO), normal plus oxidized fish oil diet (OFO), or OFO plus 0.6% (TAU1), 0.9% (TAU2) or 1.2% (TAU3) taurine diet for 10 weeks. TAG, triacylglycerol; CHOL, cholesterol; LDL-C, low-density lipoprotein cholesterol; HDL-C, high-density lipoprotein cholesterol.

**Table 4:** Serum fatty acid profile in all experimental groups of mice ^1^.

Items	CON	FFO	OFO	TAU1	TAU2	TAU3
C14:0	0.62 ± 0.01	0.52 ± 0.03	0.47 ± 0.01	0.54 ± 0.01	0.51 ± 0.01	0.43 ± 0.01
C16:0	24.99 ± 0.03	25.26 ± 1.20	29.47 ± 0.41 *	27.92 ± 1.18	28.09 ± 0.44	28.18 ± 0.79
C16:1	1.48 ± 0.06	1.16 ± 0.17 *	1.09 ± 0.05 *	1.00 ± 0.06	0.98 ± 0.03	0.99 ± 0.05
C17:0	0.67 ± 0.01	0.74 ± 0.15	0.76 ± 0.05	0.93 ± 0.06	0.86 ± 0.05	0.89 ± 0.02
C18:0	11.11 ± 0.02	7.00 ± 0.61	11.45 ± 0.89	12.64 ± 0.63	12.31 ± 0.42	12.75 ± 0.45
C18:1n9c	8.72 ± 0.10	7.91 ± 0.46	8.97 ± 0.31	8.05 ± 0.16 ^#^	8.60 ± 0.01	9.20 ± 0.26
C18:2n6c	19.56 ± 0.33	13.99 ± 1.33 *	19.05 ± 0.51	17.58 ± 0.63	17.06 ± 0.03 ^#^	16.05 ± 0.41 ^#^
C18:3n3	0.43 ± 0.03	0.67 ± 0.00 *	0.48 ± 0.00	0.30 ± 0.15	0.41 ± 0.00	0.41 ± 0.01
C20:3n6	1.56 ± 0.07	2.34 ± 0.11 *	1.76 ± 0.08	2.01 ± 0.21	2.04 ± 0.04	2.30 ± 0.06 ^#^
C20:4n6	6.87 ± 0.10	4.66 ± 0.35 *	6.16 ± 0.40	4.86 ± 0.61	5.04 ± 0.29	4.40 ± 0.25
C24:0	8.33 ± 0.27	8.98 ± 0.36	5.46 ± 0.31 *	5.18 ± 0.41	4.75 ± 0.03	4.19 ± 0.33 ^#^
C22:6n3	15.65 ± 0.18	26.11 ± 0.99 *	16.50 ± 0.23	18.98 ± 0.39 ^#^	19.33 ± 0.14 ^#^	20.17 ± 0.17 ^#^
SFA ^2^	45.73 ± 0.34	42.48 ± 0.77 *	47.62 ± 1.03	47.20 ± 0.66	46.53 ± 0.07	46.46 ± 0.53
UFA ^3^	54.27 ± 0.34	56.61 ± 0.35 *	53.00 ± 0.96	52.77 ± 0.63	53.47 ± 0.07	53.51 ± 0.51
PUFA ^4^	44.07 ± 0.29	47.54 ± 0.59 *	42.95 ± 0.60	43.73 ± 0.67	43.89 ± 0.09	43.32 ± 0.71
n-3 ^5^	17.64 ± 0.14	28.89 ± 1.21 *	21.24 ± 0.30	21.29 ± 0.40	21.79 ± 0.17	22.87 ± 0.15
n-6 ^6^	27.98 ± 0.50	20.99 ± 0.89 *	25.97 ± 0.83	24.45 ± 0.34 ^#^	24.14 ± 0.23 ^#^	22.74 ± 0.53 ^#^
SFA/UFA	0.84 ± 0.01	0.75 ± 0.02 *	0.90 ± 0.03	0.89 ± 0.02	0.87 ± 0.00	0.87 ± 0.02
n-6/n-3	1.59 ± 0.04	0.73 ± 0.06 *	1.27 ± 0.06 *	1.15 ± 0.01 ^#^	1.11 ± 0.02 ^#^	1.00 ± 0.02 ^#^

* *p* < 0.05 vs. the CON group and # *p* < 0.05 vs. the OFO group. ^1^ ICR mice were fed a normal (CON), normal plus fish oil diet (FFO), normal plus oxidized fish oil diet (OFO), or OFO plus 0.6% (TAU1), 0.9% (TAU2) or 1.2% (TAU3) taurine diet for 10 weeks. ^2^ SFA = C14:0+ C16:0+ C17:0+ C18:0+ C24:0. ^3^ UFA = C16:1+ C18:1n9c+ C18:2n6c+ C18:3n3+ C20:3n6+ C20:4n6+ C22:6n3. ^4^ PUFA = C18:2n6c+ C18:3n3+ C20:3n6+ C20:4n6+ C22:6n3. ^5^ n-3 = C18:3n3+ C22:6n3. ^6^ n-6 = C18:2n6c+ C20:3n6+ C20:4n6.

## Data Availability

Data is contained within the article.

## Supplementary Materials

The following supporting information can be downloaded at: https://www.mdpi.com/article/10.3390/antiox11071391/s1. Figure S1: The body weight of all groups of mice.
