# Supplementary material for: Taurine Attenuates Oxidized Fish Oil-Induced Oxidative Stress and Lipid Metabolism Disorder in Mice
Source: Antioxidants (Basel). 2022 Jul 18;11(7):1391. doi: 10.3390/antiox11071391 (PMC9311513; doi:10.3390/antiox11071391)
Supplement: Supplementary file 1 [file antioxidants-11-01391-s001.zip › antioxidants-1764343-SI.pdf]

antioxidants

MDPI

Supplementary Materials: Taurine Attenuates Oxidized Fish Oil-Induced Oxidative Stress and Lipid Metabolism Disorder in Mice

Qiuping Guo [1], Lingyu Zhang [1,2], Yunju Yin [1,3], Saiming Gong [1,3], Yuhuan Yang [1,3], Sisi Chen [1,4], Mengmeng Han [1,4] and Yehui Duan [1,*]

1 Hunan Provincial Key Laboratory of Animal Nutritional Physiology and Metabolic Process, CAS Key Laboratory of Agro-Ecological Processes in Subtropical Region, National Engineering Laboratory for Pollution Control and Waste Utilization in Livestock and Poultry Production, Institute of Subtropical Agriculture, Chinese Academy of Sciences, Changsha 410125, China; guoqiuping@isa.ac.cn (Q.G.); zzzhanglingyu@163.com (L.Z.); yinyj1124@163.com (Y.Y.); 15197442484@163.com (S.G.); yangyu709842339@163.com (Y.Y.); 13574350026@163.com (S.C.); 15591808189@163.com (M.H.)
2 National Engineering Laboratory for Rice and By-Product Deep Processing, Central South University of Forestry and Technology, Changsha 410004, China
3 College of Animal Science and Technology, Hunan Agricultural University, Changsha 410128, China
4 College of Advanced Agricultural Science, University of Chinese Academy of Sciences, Beijing 100039, China
* Correspondence: duanyehui@isa.ac.cn; Tel.: +86-0731-84619767

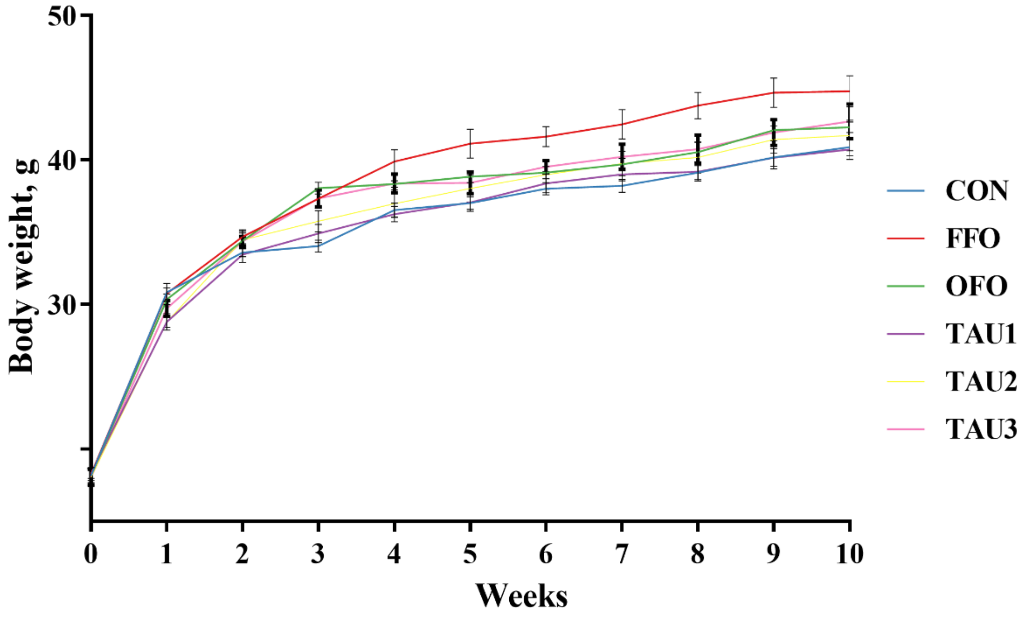

Figure S1. The body weight of all groups of mice. ICR mice were fed a normal (CON), normal plus fish oil diet (FFO), normal plus oxidized fish oil diet (OFO), or OFO plus 0.6% (TAU1), 0.9% (TAU2) or 1.2% (TAU3) taurine diet for 10 weeks.
